# Additive Value of EBUS-TBNA for Staging Non-Small Cell Lung Cancer in Patients Evaluated for Stereotactic Body Radiation Therapy

**DOI:** 10.3390/diagnostics15172136

**Published:** 2025-08-24

**Authors:** Joshua M. Boster, S. Michael Goertzen, Paula V. Sainz, Macarena R. Vial, Jhankruti K. Zaveri-Desai, Luis D. Luna, Anum Waqar, Horiana B. Grosu, Roberto F. Casal, Carlos A. Jimenez, David E. Ost, Bruce F. Sabath, Julie Lin, Mike Hernandez, Georgie A. Eapen

**Affiliations:** 1 Interventional Pulmonology Section, Department of Medicine, University of Texas MD Anderson Cancer Center, Houston, TX 77030, USA; smgoertzen@mdanderson.org (S.M.G.); pvsainz@mdanderson.org (P.V.S.); jzaveri@mdanderson.org (J.K.Z.-D.); ldluna@mdanderson.org (L.D.L.); awaqar@mdanderson.org (A.W.); hbgrosu@mdanderson.org (H.B.G.); rfcasal@mdanderson.org (R.F.C.); cajimenez@mdanderson.org (C.A.J.); dost@mdanderson.org (D.E.O.); bsabath@mdanderson.org (B.F.S.); jlin5@mdanderson.org (J.L.); geapen@mdanderson.org (G.A.E.); 2 Clínica Alemana de Santiago, Universidad del Desarrollo, Santiago 7610658, Chile; macarena.r@gmail.com; 3 Department of Biostatistics, University of Texas MD Anderson Cancer Center, Houston, TX 77030, USA; mhernandez@mdanderson.org

**Keywords:** lung cancer, stereotactic body radiation therapy, bronchoscopic ultrasound, endobronchial ultrasound, staging

## Abstract

**Background/Objectives**: Patients with non-small cell lung cancer (NSCLC) being evaluated for stereotactic body radiation therapy (SBRT) are frequently staged non-invasively with positron emission tomography/computed tomography (PET/CT). Performing endobronchial ultrasound-guided transbronchial needle aspiration (EBUS-TBNA) in addition to PET/CT scanning may increase clinical certainty in lymph node staging, but the magnitude of added benefit of EBUS-TBNA over non-invasive staging methods is unclear. **Methods**: A single-center prospective cohort study involving patients with suspected or confirmed Stage I or IIa NSCLC referred for EBUS-TBNA prior to SBRT was performed. The primary outcome was concordance between PET/CT and EBUS-TBNA for nodal metastases. Secondary endpoints included sensitivity, specificity, positive predictive value (PPV), and negative predictive value (NPV) of PET/CT, and clinical outcomes based on staging results. **Results**: Among 115 patients, the concordance between PET/CT and EBUS-TBNA was 84.3% (95% CI: 0.76 0.90). EBUS-TBNA led to a stage shift in 15.7% of cases: 4 of 98 PET/CT N0 patients (4.1%) had nodal metastases, while 14 of 17 PET/CT N1 patients (82.4%) were downstaged to N0. PET/CT sensitivity was 42.9% (95% CI: 0.09–0.81), specificity 87% (95% CI: 0.79–0.93), PPV 17.6% (95% CI: 0.04–0.43), and NPV 95.9% (95% CI: 0.90–0.99). PET/CT-positive, EBUS-TBNA-negative patients had worse survival (HR 4.25, 95% CI: 1.24–14.53, *p* = 0.021) compared with double-negative patients. **Conclusions**: EBUS-TBNA improves staging accuracy over PET/CT in early-stage NSCLC, impacting SBRT candidacy. However, PET/CT-positive, EBUS-TBNA-negative patients had worse outcomes in comparison to double-negative patients, suggesting a need for additional therapy or surveillance in that population.

## 1. Introduction

Stereotactic body radiation therapy (SBRT) has emerged as an effective therapeutic strategy for early-stage non-small cell lung cancer (NSCLC), with response rates exceeding 90% [1,2]. As the median age of lung cancer patients increases, SBRT has become particularly important for patients who are either medically inoperable or prefer a non-surgical treatment option.

Despite excellent tumor response rates, disease-free survival (DFS) is low, with some studies reporting DFS of 48% at 3 years, with out-of-field nodal and distant metastases as the most common sites of recurrence [3,4]. Low DFS rates in this population may be partially explained by the pre-treatment presence of occult lymph node metastases. Patients undergoing evaluation for SBRT are frequently staged non-invasively with positron emission tomography/computed tomography (PET/CT) alone, which may not be ideal. Prior studies have shown that nodal metastases can be present in up to 22% of patients without evidence of nodal disease on PET/CT [5]. Furthermore, PET/CT may also yield false-positive results, with rates ranging between 6% and 63% depending on the study, particularly in regions where endemic granulomatous disease is common [6,7,8]. More accurately identifying patients with nodal disease prior to treatment may improve patient selection for SBRT, which, in turn, may improve patient outcomes.

To enhance staging accuracy, some centers have incorporated endobronchial ultrasound-guided transbronchial needle aspiration (EBUS-TBNA) into their pre-SBRT evaluation protocol. While EBUS-TBNA has demonstrated superior diagnostic accuracy compared with imaging alone in patients with radiographic evidence of nodal metastases, its added benefit in SBRT-eligible patients without radiographic evidence of nodal metastases remains unclear [9]. This study aims to evaluate the clinical benefit of EBUS-TBNA in addition to PET/CT for mediastinal staging in patients with non-small cell lung cancer undergoing evaluation for SBRT.

## 2. Materials and Methods

After obtaining IRB approval (protocol 2015-0615), a single-center prospective cohort study of patients with suspected or confirmed early-stage NSCLC who were being considered for SBRT and were referred to the interventional pulmonary service at the University of Texas MD Anderson Cancer Center for EBUS-TBNA was performed between 2016 and 2023. The primary outcome was the concordance of PET/CT and EBUS-TBNA for the presence of nodal metastases. Secondary endpoints included the sensitivity, specificity, positive predictive value, and negative predictive value of PET/CT for detecting nodal metastases, with EBUS-TBNA being considered the reference standard. Clinical endpoints, including overall survival, recurrence-free survival, and locoregional recurrence rate, based on the presence or absence of nodal metastases on PET/CT and EBUS/TBNA, were also assessed.

Patients with confirmed or suspected early-stage NSCLC undergoing EBUS-TBNA as part of their routine staging workup prior to planned SBRT were included in this study. Patients were included if they were potential candidates for SBRT, independent of the final treatment. Eligible participants were over 18 years old and had proven or suspected NSCLC at clinical Stage I or IIa (T1 or T2a, N0 or N1, M0), according to the 7th edition of the American Joint Commission on Cancer (AJCC) staging system [10]. They were required to have undergone a PET/CT within 40 days prior to EBUS-TBNA, and written informed consent was obtained from the patient or their legally authorized representative before enrollment. Patients with a prior history of invasive cancer were included if they had received curative treatment and had no evidence of recurrent disease for at least 5 years. Patients were excluded if they had received prior chemotherapy or radiotherapy for their current malignancy or were already scheduled to receive conventional radiotherapy, chemotherapy, biological therapy, vaccine therapy, or surgery. Those with malignancies consistent with neuroendocrine (carcinoid) histology or those planning to undergo treatment at a different institution were also excluded from the study.

Patients meeting eligibility requirements were prospectively screened and enrolled consecutively. A lesion was classified as positive on PET/CT if the standardized uptake value (SUV) was ≥2.5. Central tumor was defined as located within the inner third of the hemithorax, and peripheral if within the outer two-thirds. Patients were considered to have a recurrence if there was a biopsy consistent with recurrence, or if imaging demonstrated evidence of progressive soft-tissue abnormalities over time that corresponded to FDG avid (SUV > 5) areas on PET/CT at least 6 months after SBRT; biopsy confirmation was not required in that circumstance. Time to recurrence was defined as the period from the completion of treatment to the earliest detection of recurrence, based on follow-up imaging or biopsy results.

EBUS-TBNA was performed under general anesthesia in the standard fashion as follows: a systematic examination of the accessible intra-thoracic lymph nodes using a linear array ultrasound bronchoscope was performed to identify those who met any of the following criteria for sampling: short axis diameter >0.5 cm and/or a combination of features associated with malignancy (sharp margins, heterogeneity, central necrosis sign, absence of a central hilar structure and rounded shape). The size of the lymph node on ultrasound imaging was measured on static images. Nodal sampling began at the contralateral hilum to avoid the possibility of specimen cross-contamination and potential staging inaccuracies. For each node, a minimum of 3 passes were performed using a 22-gauge needle. Rapid on-site cytologic evaluation (ROSE) was utilized for all procedures. The lymph node stations were described according to the International Association for the Study of Lung Cancer classification system [11].

SBRT was administered in 4 fractions over consecutive days if the total dose was 50 gray (Gy) or in 10 fractions if the total dose was 70 Gy. Patients were followed routinely, which at our institution included a physical exam and chest CT every 3 months for the first 2 years. Each patient included in this study was followed for a minimum of 2 years post-enrollment. For follow-up information, we obtained data from the medical record or contacted patients directly if necessary.

Descriptive statistics, including the mean, standard deviation, median, and range, were used to summarize continuous variables, as well as frequency count and percentage for summarizing categorical variables. Wilcoxon rank sum tests were used to compare continuous variables between study groups of interest. Chi-squared tests, or Fisher’s exact test if more appropriate, were used to compare categorical variables. Concordance between EBUS-TBNA and PET/CT lymph node staging was assessed by comparing the detection of nodal metastases between tests. Results were concordant if EBUS-TBNA did not alter nodal staging based on the 7th edition AJCC staging system (10). Discordance was defined as a change in stage in either direction following EBUS-TBNA. Other features of diagnostic accuracy such as sensitivity, specificity, positive predictive value, and negative predictive value were estimated with 95% confidence intervals. Overall survival (OS) was defined as the time from the date of diagnosis to the date of death or last contact. Participants who remained alive or were lost to follow-up were right-censored at their last observed time point, either the study endpoint or the date of last contact, respectively. Recurrence-free survival (RFS) was defined as the time from the date of diagnosis until the date of first recurrence or death due to any cause, whichever was observed first. The date the EBUS-TBNA was performed was utilized as the zero point for the survival analysis. Kaplan–Meier curves were used to provide survival estimates at select time points, and the log-rank test was used to compare time to event distributions between patient subgroups of interest for both OS and RFS. *p*-values of <0.05 were considered statistically significant. All analyses were performed using Stata v18 (StataCorp LLC, College Station, TX, USA) and R version 4.1.2 (1 November 2021).

## 3. Results

A total of 122 patients were screened and enrolled in this study, 7 of whom were excluded after enrollment, leaving 115 patients included in the analysis. Of the 7 excluded patients, 3 ultimately did not have a malignant diagnosis, and the remaining 4 were determined to be screen failures post-enrollment. Patient demographics and tumor characteristics are shown in Table 1. Most patients included in this study were white, *n* = 103 (89.6%), with a mean age of 72.3 ± 7.6 years. Mean tumor size was 2.1 ± 0.9 cm, and *n* = 89 (77.4%) were peripherally located, with the most common tumor histology being adenocarcinoma, *n* = 86 (74.8%). According to radiographic criteria, 98 (85.2%) patients had clinical N0 disease, while 17 (14.8%) had clinical N1 disease.

On EBUS-TBNA, a total of 384 lymph node stations were biopsied in 115 patients, with a mean of 3.6 ± 1.45 lymph nodes sampled per patient. Adequate samples were obtained in 95.6% of sampled lymph nodes. There were no immediate procedural complications noted.

The concordance between PET/CT and EBUS-TBNA was 84.3% (95% CI: 76.4–90.4%) (Table 2). Compared with PET/CT, EBUS-TBNA led to a stage shift in 18 out of 115 patients (15.7%). Out of 98 N0 patients by PET/CT, 4 (4.1%) had nodal metastases on EBUS-TBNA. All 4 of these patients were upstaged to N1 disease by EBUS-TBNA and subsequently received chemoradiation. Among 17 N1 patients by PET/CT, 14 (82.4%) were downstaged to N0 by EBUS-TBNA and subsequently received SBRT, while the 3 remaining patients were confirmed to have N1 disease on EBUS and received chemoradiation. The sensitivity and specificity for the presence of nodal metastases by PET/CT were 42.9% (95% CI: 0.09–0.81) and 87% (95% CI: 0.79–0.93), respectively. The positive and negative predictive values were 17.6% (95% CI: 0.04–0.43) and 95.9% (95% CI: 0.90–0.99), respectively.

**Figure 1:**
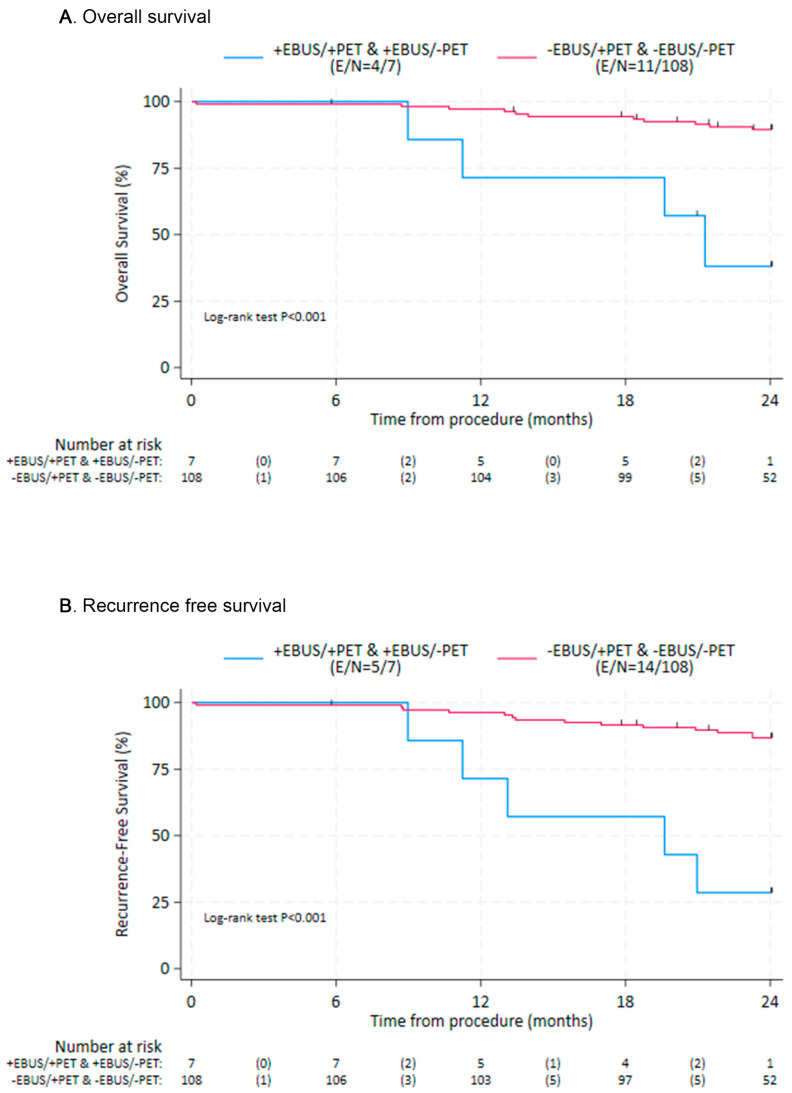
Overall survival (**A**) and recurrence-free survival (**B**) by the presence or absence of nodal metastases by EBUS-TBNA. EBUS—Endobronchial Ultrasound; PET: Positron Emission Tomography; E—Events; N—number at risk.

**Figure 2:**
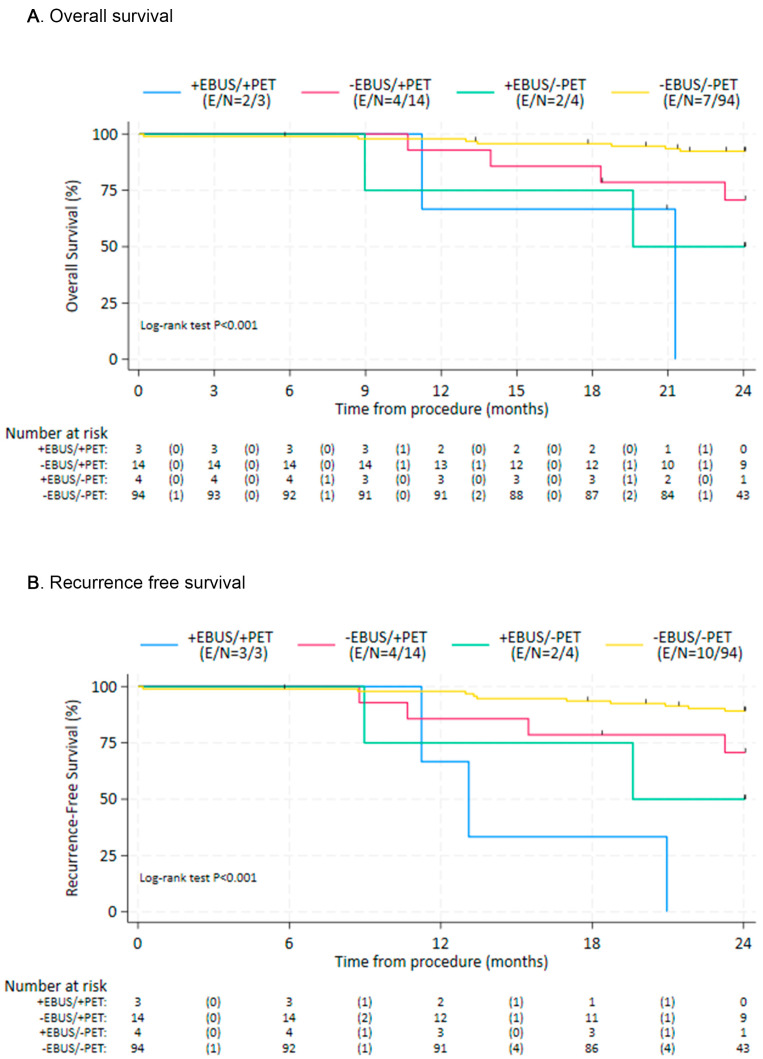
Overall survival (**A**) and recurrence-free survival (**B**) by individual study cohort. EBUS—Endobronchial Ultrasound; PET: Positron Emission Tomography; E—Events; N—number at risk.

To compare clinical outcomes, the patients were subdivided into cohorts with cohort A consisting of patients with evidence of LN metastases on both EBUS-TBNA and PET/CT (*n* = 3), cohort B negative EBUS-TBNA and positive PET/CT (*n* = 14), cohort C positive EBUS-TBNA and negative PET/CT (*n* = 4), and cohort D negative EBUS-TBNA and negative PET/CT (*n* = 94). Patients without EBUS-TBNA evidence of nodal metastases (cohorts B/D), regardless of PET/CT results, showed improved OS and RFS compared with those with a positive EBUS-TBNA (cohorts A/C), with hazard ratios (HR) = 0.13 (95% CI: 0.04, 0.42; *p* = 0.001) and HR = 0.12 (95% CI: 0.04, 0.34 *p* = 0.001), respectively (Figure 1). When using cohort D as the reference cohort, cohort B also had worse survival outcomes (Table 3). Similarly, recurrence-free survival seemed worse for cohort B in comparison to D, although the difference was not statistically significant (Table 4). Overall and recurrence-free survival outcomes were most favorable for cohorts B and D, and least favorable for cohorts A and C (Figure 2).

## 4. Discussion

Our study demonstrates that the concordance between clinical staging with PET/CT and pathological staging with EBUS-TBNA in patients with early-stage NSCLC who are being evaluated for SBRT is only moderate (84.3%). This finding is consistent with a prior retrospective study in a similar population of patients, which reported concordance between PET/CT and EBUS-TBNA of 83% [12]. The sensitivity of PET/CT for detecting nodal metastases in our study was poor (42.9%), which underscores the inadequacy of PET/CT for detecting occult nodal metastases, which may contribute to higher disease recurrence rates after SBRT compared with surgical management in early-stage NSCLC. The positive predictive value (PPV) of PET/CT was also poor (17.6%), which is highlighted by the fact that 82.4% of patients with N1 disease on PET/CT were downstaged to N0 following EBUS-TBNA. These findings suggest that reliance on PET/CT alone for staging may lead to suboptimal management decisions, emphasizing the importance of incorporating EBUS-TBNA for more accurate nodal staging in this patient population.

EBUS-TBNA was used as the reference standard for determining the sensitivity, specificity, PPV, and NPV of PET/CT in our study. Li et al. performed a retrospective study in a similar population of patients with early-stage NSCLC, who had a PET/CT preoperatively and ultimately underwent a lobectomy/systemic lymph node dissection and found that the sensitivity, specificity, PPV, and NPV of PET/CT were 74.2%, 73.2%, 54.4%, and 86.8%, respectively [13]. In our study, when EBUS-TBNA was used as the reference standard, the sensitivity and PPV of PET/CT were significantly lower (42.9% and 17.6% respectively), although the specificity and negative predictive value were comparable. The significantly lower PPV of PET/CT observed in our study may be explained by differences in patient populations with varying burdens of granulomatous disease and the use of EBUS-TBNA as the reference standard. Unlike surgical lymph node dissection, which comprehensively assesses the entire lymph node for metastases, EBUS-TBNA can miss nodal metastases due to sampling limitations, lowering the PPV of PET/CT when EBUS-TBNA serves as the reference standard. Notably, only 7 patients in our study had a positive EBUS-TBNA, with 4 being upstaged; therefore, the differences in test performance between the two studies may stem from chance alone due to the small sample size.

As expected, our study demonstrated that when lymph node metastases are confirmed by EBUS-TBNA (cohorts A/C), survival and recurrence outcomes are worse, which is consistent with prior evidence [14]. Prior studies have shown that when PET/CT alone is used to stage early-stage NSCLC patients prior to SBRT, the 2-year recurrence rate ranges from 14% to 27% [4,15]. In our study, cohort D (negative PET/CT and negative EBUS-TBNA) had a recurrence rate of 10.6%, which demonstrates the value of performing EBUS in this population when the PET/CT is negative. Interestingly, cohort B, which was the population of patients with a positive PET/CT and negative EBUS-TBNA, had worse survival outcomes compared with cohort D (negative PET/CT and negative EBUS-TBNA). This finding raises important considerations regarding the prognostic value of PET/CT positivity in the absence of nodal metastasis confirmation by EBUS-TBNA. While a negative EBUS-TBNA generally suggests the absence of nodal involvement, the worse survival outcomes observed in cohort B indicate that patients with a positive PET/CT and a negative EBUS-TBNA may harbor occult metastatic disease that was missed by EBUS-TBNA due to sampling limitations. This is consistent with prior studies suggesting that PET/CT positivity, even in the absence of pathologic confirmation, may be a marker of poorer prognosis [16,17]. Given this finding, future studies are needed to assess whether patients with a positive PET/CT and negative EBUS-TBNA warrant additional adjuvant treatment or surveillance to improve their outcomes following SBRT.

The strengths of our study include its prospective design, systematic EBUS-TBNA sampling, and the inclusion of a well-defined cohort of patients undergoing evaluation for SBRT. However, our study was performed at a single center, where all procedures were completed by highly experienced interventional pulmonologists with the availability of rapid onsite cytology, which may limit the generalizability of our findings. Furthermore, the small sample size, particularly after stratifying cohorts by PET/CT and EBUS-TBNA findings, limits the strength of our conclusions and reduces statistical power, necessitating caution in interpreting the analyses.

## 5. Conclusions

In patients with clinically early-stage NSCLC, the concordance between clinical staging with PET/CT and pathological staging with EBUS-TBNA was only moderate, and the sensitivity and positive predictive value of PET/CT alone for detecting nodal metastases were suboptimal. The incorporation of EBUS-TBNA as part of the staging evaluation for this population was beneficial in identifying occult nodal disease that would have been untreated with SBRT and in downstaging clinical N1 patients, thereby expanding the pool of patients eligible for SBRT. That said, patients with a positive PET/CT but negative EBUS-TBNA had worse outcomes in comparison to patients who were negative on both EBUS-TBNA and PET/CT, raising the question as to the utility of adjuvant therapy or additional surveillance in such patients. Future studies are needed to better assess whether the routine incorporation of EBUS-TBNA staging for these patients improves clinical outcomes, with a particular focus on patients with discordant results, and should be the subject of larger prospective and randomized studies.

## Figures and Tables

**Table 1:** Demographic and tumor characteristics *n* = 115.

Age (years), mean ± SD	72.3 ± 7.6
Gender (n. %)	
F	51 (44.3%)
M	64 (55.7%)
Race (n. %)	
White	103 (89.6%)
Non-white	12 (10.4%)
Smoking hx (n. %)	
Never smoked	1 (0.9%)
Former smoker	106 (92.2%)
Current smoker	8 (6.9%)
ECOG (n. %)	
0	54 (47.0%)
1	54 (47.0%)
>2	7 (6.0%)
Pulmonary function tests (mean ± SD)	
FEV1%	79.2 ± 24.8
DLCO	80.7 ± 24.5
Tumor Size (cm), mean ± SD	2.1 ± 0.9
Tumor centrality (n. %)	
Peripheral	89 (77.4%)
Central	26 (22.6%)
Tumor Histology (n. %)	
Adenocarcinoma	86 (74.8%)
Squamous cell carcinoma	22 (19.1%)
Other malignancies	7 (6.1%)
Tumor FDG Avidity (n. %)	
SUV < 2.5	23 (20.0%)
SUV ≥ 2.5	92 (80.0%)

SD—standard deviation; *n*—number; SUV—standardized uptake value; ECOG—Eastern Cooperative Oncology Group Performance Status; cm—centimeter; F—female; M—male.

**Table 2:** Concordance Between EBUS-TBNA and PET/CT for the Detection of Nodal Metastases.

	EBUS Positive	EBUS Negative	Total	Concordance Rate (95% CI)
PET/CT Positive	3 (2.6%)	14 (12.2%)	17	84.3% (76.4%−90.4%)
PET/CT Negative	4 (3.5%)	94 (81.7%)	98	
Total	7	108	115	

EBUS—endobronchial ultrasound; PET/CT—positron emission tomography and computed tomography; CI—confidence interval.

**Table 3:** Summary of Overall Survival by Study Cohort.

Cohort	N; (Deaths)	Median (95%CI)	HR(95%CI)	*p*-Value
A	3 (2)	21.29 (11.24, NE)		
B	14 (4)	NR (18.33, NE)	4.25 (1.24, 14.53)	0.021
C	4 (2)	19.61 (8.97, NE)		
D	94 (7)	NR	Ref.	

NR—Median was not reached; NE confidence limit was not estimable.

**Table 4:** Summary of Recurrence Free Survival by Study Cohort.

Cohort	N; (Events)	Median (95%CI)	HR(95% CI)	*p*-Value
A	3 (3)	13.11 (11.24, NE)		
B	14 (4)	NR (15.47, NE)	3.06 (0.96, 9.76)	0.059
C	4 (2)	19.61 (8.97, NE)		
D	94 (10)	NR	Ref.	

NR—Median was not reached; NE confidence limit was not estimable; CI—Confidence interval; N—Number; Ref.—Reference.

## Data Availability

The data that support the findings of this study are available on request from the corresponding author.

## Abbreviations

The following abbreviations are used in this manuscript:

NSCLC	Non-small cell lung cancer
SBRT	Stereotactic body radiation therapy
PET/CT	Positron emission tomography/computed tomography
EBUS-TBNA	Endobronchial ultrasound-guided transbronchial needle aspiration
PPV	Positive predictive value
NPV	Negative predictive value
IRB	Institutional Review Board
IACUC	Institutional Animal Care and Use Committee
DFS	Disease-free survival
AJCC	American Joint Commission on Cancer
SUV	Standardized uptake value
FDG	Fluorodeoxyglucose
ROSE	Rapid onsite cytologic evaluation
Gy	Gray
OS	Overall survival
RFS	Recurrence-free survival
HR	Hazard ratio
CI	Confidence interval
cm	Centimeters
SD	Standard deviation
ECOG	Eastern Cooperative Oncology Group
FEV1	Forced expiratory volume in 1 s
DLCO	Diffusing capacity of the lung for carbon monoxide
NR	Not reached
NE	Not estimable
